# Evaluation of OCT Angiography Parameters as Biomarkers for Glaucoma Progression

**DOI:** 10.3390/diagnostics16010035

**Published:** 2025-12-23

**Authors:** Konstantina Kancheva, Mladena Radeva, Igor B. Resnick, Zornitsa Zlatarova

**Affiliations:** 1 Prof. D-r Paraskev Stojanov, Medical University Varna, 9002 Varna, Bulgaria; 2 Faculty of Medicine, University “Prof Dr Asen Zlatarov”, 8010 Burgas, Bulgaria; 3 Department of Ophthalmology, Medical University Varna, 9002 Varna, Bulgaria; 4 Department of Ophthalmology and Visual Sciences, Faculty of Medicine, Medical University, 9002 Varna, Bulgaria

**Keywords:** glaucoma, optical coherence tomography angiography (OCT-A), retinal nerve fiber layer (RNFL), vessel density, visual field, primary open-angle glaucoma, peripapillary microvasculature

## Abstract

**Background:** Optical coherence tomography angiography (OCT-A) provides quantitative assessment of retinal and peripapillary microvasculature and has emerged as a promising tool for glaucoma diagnostics. However, its sensitivity for detecting early glaucomatous progression over short intervals remains uncertain. This study evaluated cross-sectional and short-term longitudinal OCT-A vessel density (VD) metrics in primary open-angle glaucoma (POAG) and explored their relationships with structural (RNFL) and functional (MD) measures. **Methods:** Sixty eyes (30 POAG, 30 controls) underwent baseline and 6-month examinations including intraocular pressure (IOP), standard automated perimetry (SAP), structural OCT, and OCT-A (RTVue XR Avanti; AngioVue). Parameters analyzed included peripapillary VD (PP-VD), parafoveal VD (PF-VD), foveal avascular zone (FAZ) metrics, FD-300, and RNFL thickness. Between-group comparisons used *t*-tests or Mann–Whitney U tests. Effect sizes (Cohen’s d), 95% confidence intervals (CI), and ANCOVA models (adjusted for baseline, age, and sex) were included. Longitudinal change was defined as Δ = 6 months − baseline. Pearson correlations evaluated structure–vascular associations. **Results:** At baseline, POAG eyes showed significantly lower PP-VD, PF-VD, thinner RNFL, and worse MD (all *p* < 0.001). Strong correlations were observed between RNFL and PP-VD (r ≈ 0.7). Over 6 months, glaucoma eyes showed small but statistically significant reductions in RNFL (Δ = −1.04 µm), MD (Δ = −0.10 dB), and PP-VD (Δ = −0.57%), whereas controls remained stable. However, the absolute OCT-A changes were small and largely within the known range of test–retest variability. ANCOVA demonstrated a significant adjusted group effect only for PP-VD (B = −1.22%, 95% CI −1.53 to −0.90; *p* < 0.001). **Conclusions:** OCT-A demonstrated clear cross-sectional differences between POAG and controls and strong structure–vascular associations. However, with only two measurements over a 6-month interval, the study cannot distinguish true glaucomatous progression from physiological or device-related variability. Short-term changes should therefore be interpreted cautiously. PP-VD remains the most robust and consistent OCT-A parameter, but larger, longer, and prospectively powered studies are required to validate OCT-A as a reliable biomarker for progression.

## 1. Introduction

Glaucoma is a chronic, progressive optic neuropathy characterized by the loss of retinal ganglion cells (RGCs) and corresponding visual field (VF) defects, and remains one of the leading causes of irreversible blindness worldwide [1,2]. Experimental studies have demonstrated that RGC death in glaucoma is a complex process involving multiple molecular pathways, including oxidative stress–induced injury, mitochondrial dysfunction, excitotoxicity, glial activation, and apoptosis-related signaling cascades [3]. Two major pathophysiological frameworks are traditionally used to explain glaucomatous damage: the mechanical theory and the vascular theory. The mechanical theory attributes damage to intraocular pressure (IOP)–induced deformation of the lamina cribrosa and mechanical compression of RGC axons, while the vascular theory proposes that reduced ocular perfusion and microvascular dysregulation lead to ischemic injury of the optic nerve head (ONH) [4,5,6]. Increasing evidence suggests that these mechanisms are not mutually exclusive but operate in combination, with vascular insufficiency exacerbating mechanical stress and vice versa [6].

Advances in optical coherence tomography (OCT) have significantly improved the structural assessment of glaucoma, enabling reproducible measurement of the retinal nerve fiber layer (RNFL) and ganglion cell complex (GCC) [7]. However, structural loss often precedes detectable VF impairment: it is well established that up to 30–50% of RGCs may be lost before a VF defect appears, highlighting the limitations of VF testing as a subjective, variable, and fatigue-dependent measure, particularly in elderly patients [8]. This underscores the need for objective, quantitative biomarkers capable of detecting earlier functional and microvascular alterations.

Optical coherence tomography angiography (OCT-A) has emerged as a non-invasive, dye-free imaging modality capable of visualizing and quantifying microvascular networks in the retina and ONH. OCT-A detects motion contrast generated by erythrocytes across sequential OCT scans, producing high-resolution maps of vessel density (VD) and perfusion [9,10,11]. The split-spectrum amplitude-decorrelation angiography (SSADA) algorithm incorporated in the AngioVue system enables robust quantification of VD and flow indices [12,13].

Numerous studies have demonstrated reduced peripapillary and macular VD in glaucoma, with strong correlations to RNFL thickness, GCC loss, and VF mean deviation (MD) [14,15,16]. OCT-A has been shown to differentiate healthy, suspect, and glaucomatous eyes [11,17], characterize regional microvascular defects corresponding to structural changes [18,19,20,21], and identify focal microvascular dropout associated with lamina cribrosa abnormalities [22]. Recent advancements, including wide-field OCT-A, have further improved diagnostic accuracy and structure–function correspondence [21,23].

Growing evidence suggests that microvascular abnormalities not only reflect existing structural damage but may precede or predict glaucomatous functional loss [8,24]. Longitudinal studies have reported progressive VD decline in POAG [24,25,26], postoperative microvascular recovery after IOP-lowering surgery [22], and moderate sensitivity of OCT-A parameters for detecting temporal changes [25,26,27,28].

Despite substantial progress, it remains unclear whether OCT-A–derived microvascular parameters can reliably detect early glaucomatous change over short follow-up intervals, and how strongly these parameters relate to established structural (RNFL) and functional (MD) markers. The limited number of longitudinal studies, lack of standardized protocols, and ongoing debate regarding test–retest variability emphasize the need for additional clinical data examining the short-term behavior of OCT-A parameters in glaucoma.

The present study aimed to evaluate the diagnostic relevance of OCT-A in primary open-angle glaucoma by comparing VD metrics between POAG patients and healthy controls, and by assessing the relationships between OCT-A parameters, RNFL thickness, and VF mean deviation. Additionally, we explored short-term (6-month) changes in these parameters to evaluate whether OCT-A may reflect early microvascular trends relevant to glaucoma monitoring.

## 2. Methods

### 2.1. Participants

POAG was defined as the presence of an open iridocorneal angle, characteristic glaucomatous optic nerve head damage (i.e., neuroretinal rim thinning, notching, or an RNFL defect), and a corresponding glaucomatous VF defect.

A glaucomatous VF defect was defined as meeting one or more of the following criteria:

1. “Outside normal limits” on the Glaucoma Hemifield Test;
2. Three or more abnormal points with *p* < 0.05 and at least one with *p* < 0.01 on the pattern deviation plot; or
3. A pattern standard deviation with *p* < 0.05 confirmed on two consecutive reliable tests (fixation losses ≤ 20% and false-positive and false-negative rates ≤ 25%).

Healthy controls had an intraocular pressure (IOP) ≤ 21 mm Hg, no history of elevated IOP, a normal-appearing optic disc, and a normal visual field. When both eyes met the inclusion criteria, one eye was randomly selected for analysis.

Participants were recruited consecutively from the outpatient glaucoma clinic of the Specialized Eye Hospital for Active Treatment—Burgas, Bulgaria. All glaucoma patients included in the study were already diagnosed and registered for long-term follow-up in the hospital (dispensary patients) and were routinely monitored at regular clinical visits. No newly diagnosed POAG cases were included, ensuring that all participants were familiar with visual field and OCT testing procedures. Healthy controls were also recruited consecutively from the same institution and were frequency-matched by age. Consecutive enrollment minimized selection bias and ensured that all eligible patients presenting during the recruitment period were invited to participate.

Exclusion criteria included high refractive error (>±6.0 diopters of spherical equivalent), axial length > 26.5 mm, or any history of amblyopia. Participants with macular pathology (including age-related macular degeneration, diabetic macular edema, epiretinal membrane), significant media opacity, or any retinal or optic nerve disease other than POAG were excluded. Individuals reporting systemic vascular conditions known to affect ocular microcirculation (e.g., uncontrolled hypertension, diabetes with vascular complications, recent cardiovascular events), or those receiving unstable vasoactive medication regimens, were also excluded. This ensured that OCT-A measurements were not confounded by systemic vascular instability.

### 2.2. Ophthalmologic Examination

Before enrollment, all participants underwent a comprehensive ophthalmic examination including

- detailed ocular and systemic history;
- best-corrected visual acuity for each eye;
- Goldmann applanation tonometry;
- indirect ophthalmoscopy;
- gonioscopy with a Goldmann lens;
- pachymetry;
- SAP;
- OCT.

All examinations were repeated at the 6-month follow-up visit.

### 2.3. Standard Automated Perimetry

SAP was performed using a Humphrey Field Analyzer II perimeter (Carl Zeiss Meditec, Dublin, CA, USA), the current gold standard for glaucoma diagnosis and monitoring. Stimulus size III, program 30-2, and the Full Threshold strategy were used. Only reliable tests meeting the criteria described above were included in the analysis. To assess functional status and potential change over time, the global index MD was evaluated. All patients had previously diagnosed and followed POAG; therefore, SAP testing was familiar to all participants.

### 2.4. Optical Coherence Tomography and OCT Angiography

Structural and vascular imaging of the ONH and peripapillary retina were obtained using the RTVue XR Avanti spectral-domain OCT scanner (Optovue Inc., Fremont, CA, USA; software version 2018.1.1.63). This system performs high-resolution imaging of both the anterior and posterior eye segments.

From the ONH/GCC Summary Report, the following structural parameters were extracted: average RNFL thickness and the global loss volume (GLV).

OCT angiography was performed with the AngioVue module. The following quantitative vascular parameters were analyzed:

- peripapillary VD—PP-VD;
- parafoveal VD—PF-VD;
- foveal avascular zone (FAZ)
- FD-300 (VD in a 300-µm annulus surrounding the FAZ).

Only scans with optimal image quality (signal strength index > 50) and without motion artifacts, vitreous floaters, or segmentation errors were included. Artificial tears were instilled as needed, and subjects were instructed to blink before image acquisition to optimize the tear film.

Goldmann tonometry was performed between 2:00 p.m. and 4:00 p.m., before SAP and OCT imaging.

At baseline and after 6 months, all participants underwent a complete ophthalmic examination including SAP and OCT/OCTA measurements.

### 2.5. Justification of Follow-Up Interval

The study employed a 6-month follow-up interval because this corresponds to the standard clinical monitoring schedule for dispensary glaucoma patients in our institution, where structural and functional testing is routinely performed twice per year. Short-term intervals of similar length have also been used in previous exploratory OCT-A studies evaluating microvascular variability under real-world conditions. However, we acknowledge that a 6-month period with only two measurement time points is insufficient to characterize true glaucomatous progression, as subtle OCT-A changes may fall within physiological or device-related variability. The longitudinal findings should therefore be interpreted as preliminary trends rather than definitive evidence of progression.

### 2.6. Statistical Analysis

Data Analyses were performed using Stata v16 and Python version 3.10 (SciPy and statsmodels). Continuous variables were expressed as mean ± standard deviation (SD) or median (interquartile range), and categorical variables as counts and percentages. Normality was assessed using the Shapiro–Wilk test. Between-group comparisons were performed using the independent-samples *t*-test or the Mann–Whitney U test, as appropriate. Categorical variables were compared using the χ^2^ test or Fisher’s exact test. All primary comparisons include effect sizes and precision estimates: Cohen’s d for continuous variables, 95% confidence intervals (95% CI) for between-group differences, and partial eta-squared (η^2^p) for ANCOVA models. Longitudinal change was calculated as Δ = follow-up − baseline for all structural and vascular parameters.

Adjusted group effects were examined using analysis of covariance (ANCOVA), with the 6-month value entered as the dependent variable, baseline value as a covariate, and group (POAG vs. control), age, and sex as independent variables. This model provides adjusted group differences, their 95% CI, and associated effect size (η^2^p).

Associations between RNFL thickness, mean deviation (MD), and OCT-A parameters were evaluated using Pearson’s correlation coefficient (r). All tests were two-tailed, and statistical significance was set at *p* < 0.05.

## 3. Results

A total of 60 eyes (30 with primary open-angle glaucoma and 30 healthy controls) were included in the study. Baseline demographic, clinical, structural, and OCT-A parameters are presented in Table 1. The two groups were comparable in age and sex. As expected, glaucoma eyes showed significantly thinner RNFL, more negative MD values, and higher baseline IOP compared with controls.

1. Baseline OCT-A and Structural Parameters

**Figure 1:**
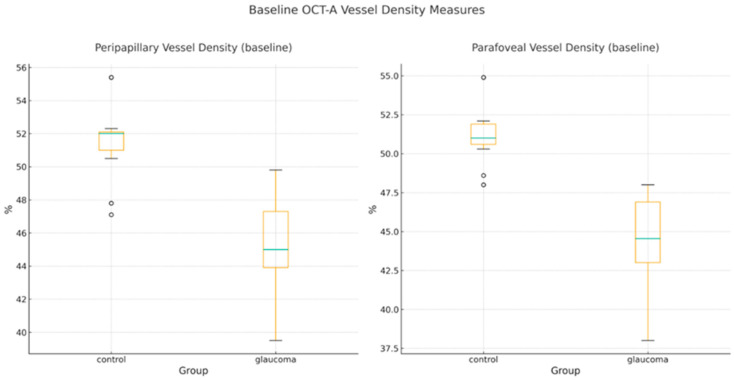
Baseline PP-VD and PF-VD in glaucoma and control eyes. Boxplots showing PP-VD and PF-VD at baseline in glaucoma and healthy control eyes. Boxes represent the interquartile range with median values; whiskers indicate non-outlier ranges. Individual data points are overlaid to illustrate within-group variability. Glaucoma eyes demonstrate consistently lower VD across both regions.

At baseline, glaucoma eyes demonstrated consistently lower VD across all OCT-A metrics (Table 1). PP-VD was reduced by 6.31% (95% CI, −7.52 to −5.10; Cohen’s d = 2.69), PF-VD by 6.67% (95% CI, −7.87 to −5.47; d = 2.89), and RPC whole-image density by 6.57% (95% CI, −7.73 to −5.41; d = 2.93). These differences are visually evident in Figure 1, which shows wider distributions and notably lower medians in glaucoma eyes.

2. Six-Month Changes in Structural and OCT-A Parameters

Six-month changes (Δ) for both groups are summarized in Table 2. Glaucoma eyes showed small but statistically significant changes over this interval.

**Figure 2:**
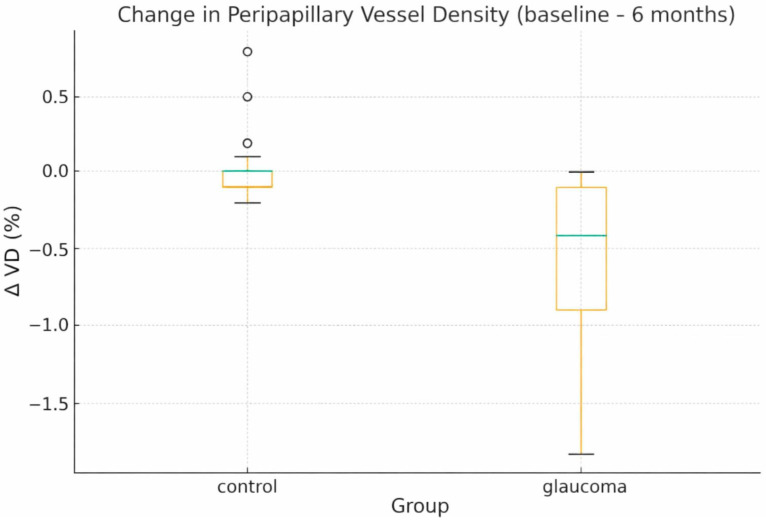
Six-month change in PP-VD in glaucoma and control eyes. Boxplots illustrating the six-month change (Δ = 6 months − baseline) in PP-VD for glaucoma and control eyes. Boxes represent the interquartile range and medians; whiskers show non-outlier values. Individual data points are plotted to demonstrate the distribution of changes. Glaucoma eyes show a small but consistent decrease in PP-VD, whereas controls remain stable.

- RNFL thickness decreased by 1.04 µm in glaucoma eyes versus 0.22 µm in controls (between-group Δ difference: −0.82 µm; 95% CI, −1.28 to −0.36; d = 0.94).
- MD declined modestly in glaucoma eyes (Δ = −0.10 dB), while controls showed slight improvement (Δ = +0.06 dB). The between-group difference of −0.16 dB (95% CI, −0.26 to −0.06; d = 0.81) was statistically significant.
- PP-VD decreased by 0.57% in glaucoma eyes, whereas controls remained essentially stable (Δ = +0.02%). The between-group Δ difference was −0.59% (95% CI, −0.81 to −0.37; d = 1.41), and individual values are depicted in Figure 2.
- Changes in PF and whole-RPC VD were more variable and did not differ significantly between groups, consistent with their broader baseline variance.

3. ANCOVA Adjusted Six-Month Outcomes

To determine whether glaucoma status independently influenced 6-month outcomes after adjusting for baseline level, age, and sex, ANCOVA models were constructed for each parameter (Table 3).

Only PP-VD at 6 months showed a significant adjusted group effect, with an estimated difference of −1.22% (95% CI, −1.53 to −0.90; F (1,55) = 58.64; *p* < 0.001; partial η^2^ = 0.516).

For RNFL thickness, the adjusted effect was borderline significant (*p* ≈ 0.05). MD, IOP, PF-VD, and whole-RPC VD did not show significant adjusted differences.

The high R^2^ values in some models reflect the strong dependence of 6-month measurements on their baseline levels—an expected phenomenon in short-interval follow-up studies with minimal biological change.

4. Relationship Between Structural and Vascular Parameters

**Figure 3:**
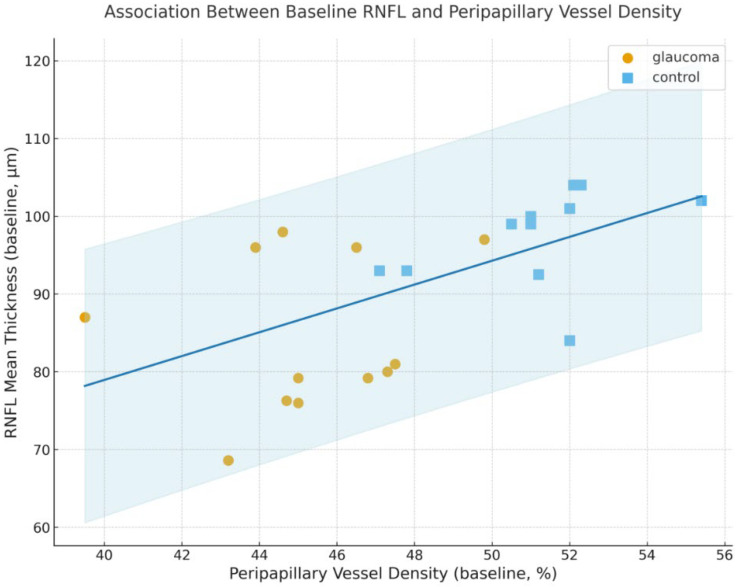
Association between baseline RNFL thickness and PP-VD Scatterplot showing the association between baseline RNFL mean thickness and PP-VD for each group. A fitted linear regression line with its 95% confidence interval is displayed. Glaucoma eyes cluster toward lower RNFL and lower PP-VD values, reflecting parallel structural and microvascular loss.

A scatterplot of baseline RNFL thickness versus baseline PP-VD (Figure 3) showed a clear positive association, with glaucoma eyes clustering in the lower-left region of the plot. Although not the primary aim of the study, this visual relationship reinforces the parallel loss of structure and microvasculature in glaucomatous damage.

## 4. Discussion

In the present study, we evaluated the diagnostic and monitoring potential of OCT-A–derived VD parameters in patients with POAG compared with healthy controls. Our results demonstrate that PP-VD and PF-VD were significantly reduced in glaucomatous eyes, correlating strongly with RNFL thickness and visual field MD. These findings are consistent with previous reports supporting the role of vascular impairment in glaucomatous optic neuropathy [4,5,6,11,14]. In particular, our observation of lower PP-VD and PF-VD in glaucoma versus controls agrees with the cross-sectional results of Yarmohammadi et al. [8,11], and Chen et al. [14], who similarly reported reduced VD in glaucomatous eyes and robust correlations with RNFL thinning and visual field loss.

The vascular theory of glaucoma suggests that reduced ocular blood flow and dysregulation of microcirculation contribute to ischemic injury of RGCs [4,5]. OCT-A allows the non-invasive quantification of these microvascular changes, offering insight into the relationship between structural and vascular damage. Our results confirm that OCT-A metrics, particularly PP-VD, are highly correlated with RNFL thickness (r ≈ 0.7, *p* < 0.001), supporting the concept of a structural–vascular coupling in glaucomatous damage. This agrees with earlier studies by Yarmohammadi et al. [11] and Chen et al. [14], who demonstrated that VD reduction parallels structural thinning and functional loss. Similarly, Yarmohammadi et al. found that PP-VD strongly reflects visual field damage [8], and Lei J et al. showed close association between PP-VD and RNFL thickness [29]. Our correlation coefficients fall within the moderate-to-strong range reported in these studies, reinforcing the idea that microvascular compromise and structural damage evolve in parallel.

The present results also align with previous reports that have investigated regional and hemifield-specific microvascular changes. Rao et al. [12] and Akagi et al. [13] described localized VD loss corresponding to structural and functional deficits, whereas Patel NB et al. [15] and Suh MH et al. [30] demonstrated that optic disc and peripapillary microvascular alterations mirror optic nerve head morphology. Although our study did not perform detailed sectoral analysis beyond global peripapillary and parafoveal indices, the overall pattern of reduced VD in glaucomatous eyes is comparable to these regional findings. Our data therefore support the broader concept that OCT-A captures both diffuse and localized microvascular changes associated with glaucomatous optic neuropathy.

However, our 6-month follow-up with only two measurement time points does not allow a reliable assessment of true glaucoma progression. Prior literature demonstrates that OCT-A measurements exhibit meaningful short-term variability driven by signal strength, motion artifacts, ocular perfusion fluctuations, and device reproducibility [29]. Studies by Park et al. [22] and Lin et al. [24] show that longitudinal VD changes are typically small and often require multi-year follow-up to exceed test–retest variability. In Park et al., postoperative microvascular recovery after glaucoma surgery was detectable but still modest over the observed period [22], and Lin et al. reported gradual microvascular decline over longer follow-up intervals [24]. Compared with these studies, our 6-month interval is shorter and our design simpler, which helps explain why the absolute changes in VD were small and must be interpreted with caution. Therefore, the subtle changes detected in our POAG group should be interpreted as exploratory observations rather than firm evidence of progression.

Moreover, recent longitudinal OCT-A research emphasizes that detecting true microvascular progression requires multiple follow-up visits and sophisticated trend-based analyses [24,25,26,27,28,30], which were beyond the scope of our study. Our findings are thus more comparable to early-phase or pilot longitudinal studies, which primarily demonstrate persistent cross-sectional differences and only modest, borderline longitudinal trends, rather than to large-scale progression trials.

The level of correlation between OCT-A metrics in our dataset (e.g., r = 0.96 between PP-VD and PF-VD) suggests redundancy among vascular parameters, consistent with previous studies showing collinearity of OCT-A metrics due to overlapping vascular networks [18,21,23]. Such collinearity limits the incremental diagnostic value of adding multiple OCT-A metrics and highlights the need to identify which parameters are most informative in clinical practice. Future multivariable analyses may clarify which OCT-A biomarkers offer independent predictive value. This issue has also been raised in recent reviews and systematic analyses [17,20,23,28], which emphasize that PP-VD often captures most of the clinically relevant information, with additional metrics providing only marginal gains. Our own findings, showing the strongest and most consistent effects for PP-VD, are in line with this view.

In our dataset, PP-VD emerged as the most stable and informative OCT-A parameter, demonstrating the strongest cross-sectional group differences and the most consistent structure–function associations. This pattern is in line with prior reports indicating that peripapillary microvascular loss most closely parallels RNFL thinning and visual field damage, likely due to the dense concentration of radial peripapillary capillaries surrounding the optic nerve head. Although the small magnitude of 6-month change in PP-VD must be interpreted cautiously and cannot be considered evidence of progression, its relative robustness compared with other vascular metrics suggests that PP-VD may hold greater clinical relevance for early detection and risk stratification. Importantly, our findings should not be extrapolated to claim longitudinal sensitivity; rather, they reinforce the notion that PP-VD may be a promising parameter for future, adequately powered multi-time-point studies aiming to evaluate progression.

From a clinical perspective, OCT-A offers a complementary perspective to structural OCT and functional testing. Diagnostic studies have shown strong performance of VD in differentiating healthy from glaucomatous eyes [8,17,18,19,21], and OCT-A has been proposed as a useful adjunct in early or uncertain cases. For example, Akil et al. and Javed, A et al. highlighted that OCT-A may improve detection of early disease and glaucoma suspects when combined with RNFL and GCC measurements [17,28]. Hong RK et al. further extended this concept using wide-field OCT-A, demonstrating good diagnostic ability in more peripheral regions [21]. Our results, showing clear cross-sectional differences in PP-VD and PF-VD and strong structure–function correlations, are consistent with these reports and support the role of OCT-A as an adjunct rather than a replacement for established structural and functional tests. However, limitations including segmentation artifacts, motion-induced error, signal-strength dependence, and reduced reproducibility in deeper layers must be considered [29]. Systematic reviews by Bekkers et al. and others [20,23] have stressed that these technical factors, together with inter-device and inter-software variability, can influence quantitative measurements and must be accounted for when interpreting OCT-A data in both research and clinical practice.

Our study has several important limitations. The sample size was modest, and no a priori power calculation was performed, which limits the ability to detect small but clinically meaningful longitudinal changes. The short follow-up interval (6 months) and the use of only two measurement time points further restrict the interpretation of temporal trends, as prior work has shown that OCT-A VD changes over such short periods often fall within the range of physiological fluctuation and test–retest variability. Consequently, the slight structural and microvascular differences observed here should be considered exploratory and not definitive evidence of glaucomatous progression. Although we adjusted for age and sex, systemic vascular factors (e.g., blood pressure, cardiovascular disease, vasoactive medications) were not incorporated into the models and may influence microvascular measurements. Compared with larger multicenter cohorts reported in the literature [8,20,24], the present study should therefore be regarded as a single-center pilot analysis. Future studies with larger, prospectively powered samples and multiple follow-up time points are required to validate the longitudinal sensitivity and clinical utility of OCT-A–derived parameters in glaucoma progression.

Despite these limitations, the present study contributes to growing evidence that OCT-A–derived VD provides clinically relevant information on glaucomatous damage. The technology’s non-invasive, repeatable, and quantitative nature makes it an attractive tool for both clinical practice and research applications. By demonstrating that PP-VD remains the most robust and consistent parameter in a short-term, real-world clinical cohort, and by explicitly situating our findings within the context of recent cross-sectional, longitudinal, and review studies [8,17,18,19,20,21,22,23,24,25,26,27,28,29,30], our work adds nuance to the ongoing discussion regarding the strengths and limitations of OCT-A as a biomarker in glaucoma.

## 5. Conclusions

This study confirms that OCT-A detects significant cross-sectional microvascular impairment in POAG, with robust reductions in PP-VD and PF-VD and strong correlations with RNFL thickness and visual field loss. These findings reinforce the established structural–vascular relationship in glaucoma and support the diagnostic value of OCT-A as an adjunct to conventional OCT and perimetry.

However, the short 6-month follow-up and the presence of only two measurement time points impose critical limitations on the longitudinal interpretation of our data. The modest OCT-A and RNFL changes observed in this interval fall near the expected range of physiological fluctuation and device-related variability, meaning they cannot be interpreted as confirmed glaucomatous progression. As such, our longitudinal results should be regarded as exploratory trends rather than definitive evidence of temporal microvascular decline.

Despite the limitations of our study, PP-VD consistently emerged as the most reliable and reproducible OCT-A parameter and may hold promise for future progression monitoring. Larger, multisite studies with standardized acquisition protocols, extended follow-up intervals, and trend-based analyses are needed to establish the clinical utility of OCT-A as a biomarker of early glaucomatous progression.

## Figures and Tables

**Table 1:** Baseline demographic, structural, functional, and OCT-A parameters in glaucoma and control eyes.

Variable	Glaucoma (n = 30)	Control (n = 30)	Between-Group Diff (G−C)	95% CI	Cohen’s d	*p*-Value
Age (years)	65.77 ± 3.94	64.87 ± 5.64	0.90	−1.62 to 3.42	0.18	0.477
RNFL mean (baseline, µm)	85.90 ± 10.03	98.02 ± 6.11	−12.12	−16.43 to −7.81	−1.46	8.54 × 10^−7^
MD (baseline, dB)	−2.82 ± 0.48	0.64 ± 0.85	−3.45	−3.81 to −3.10	−5.01	8.91 × 10^−24^
IOP (baseline, mmHg)	19.28 ± 1.16	16.48 ± 1.68	2.80	2.06 to 3.55	1.94	7.45 × 10^−10^
PP-VD (%)	45.33 ± 2.74	51.64 ± 1.85	−6.31	−7.52 to −5.10	−2.69	3.02 × 10^−14^
PF-VD (%)	44.58 ± 2.84	51.25 ± 1.62	−6.67	−7.87 to −5.47	−2.89	9.93 × 10^−15^
RPC VD whole image (%)	43.69 ± 2.66	50.27 ± 1.71	−6.57	−7.73 to −5.41	−2.93	2.13 × 10^−15^

**Table 2:** Six-month changes (Δ) in structural, functional, and OCT-A parameters.

Variable	Glaucoma (n = 30)	Control (n = 30)	Δ Difference (G–C)	95% CI	Cohen’s d	*p*-Value
Δ RNFL mean (µm)	−1.04 ± 0.40	−0.22 ± 1.16	−0.82	−1.28 to −0.36	−0.94	0.000828
Δ MD (dB)	−0.10 ± 0.24	+0.06 ± 0.14	−0.16	−0.26 to −0.06	−0.81	0.00281
Δ IOP (mmHg)	−0.21 ± 1.40	+0.42 ± 0.81	−0.63	−1.22 to −0.04	−0.55	0.0379
Δ PP-VD (%)	−0.57 ± 0.56	+0.02 ± 0.21	−0.59	−0.81 to −0.37	−1.41	3.37 × 10^−6^
Δ PF-VD (%)	−0.15 ± 0.59	−0.54 ± 1.49	+0.39	−0.20 to 0.99	0.35	0.187
Δ RPC whole-image VD (%)	−0.35 ± 0.58	+0.00 ± 0.42	−0.35	−0.62 to −0.09	−0.69	0.00997

**Table 3:** ANCOVA for 6-month outcomes adjusted for baseline, age, and sex.

Outcome	Group Effect B (G vs. C)	95% CI for B	F (1,55)	*p*-Value	Partial η^2^	Model R^2^	Adj. R^2^
RNFL mean (6 months, µm)	−0.56	−1.13 to 0.00	4.00	0.0505	0.068	0.994	0.993
MD (6 months, dB)	−0.20	−0.48 to 0.08	2.05	0.157	0.036	0.991	0.990
IOP (6 months, mmHg)	+0.43	−0.35 to 1.20	1.23	0.272	0.022	0.679	0.656
PP-VD (6 months, %)	−1.22	−1.53 to −0.90	58.64	<0.001	0.516	0.993	0.992
PF-VD (6 months, %)	−0.59	−1.62 to 0.44	1.30	0.258	0.023	0.924	0.918
RPC whole-image VD (6 months, %)	+0.11	−0.33 to 0.55	0.26	0.610	0.005	0.989	0.988

## Data Availability

Data are available on request from the corresponding author.
